# Melatonin regulates the aging mouse hippocampal homeostasis via the sirtuin1-FOXO1 pathway

**DOI:** 10.17179/excli2016-852

**Published:** 2017-03-23

**Authors:** Anorut Jenwitheesuk, Parichart Boontem, Prapimpun Wongchitrat, Jiraporn Tocharus, Sujira Mukda, Piyarat Govitrapong

**Affiliations:** 1 Research Center for Neuroscience, Institute of Molecular Biosciences, Mahidol University, Salaya, Nakornpathom, Thailand; 2 Chulabhorn Graduate Institute, Chulabhorn Royal Academy, Bangkok 10210, Thailand; 3 Center for Research and Innovation, Faculty of Medical Technology, Mahidol University, Salaya, Nakon Pathom 73170, Thailand; 4 Department of Physiology, Faculty of Medicine, Chiang Mai University, Chiang Mai 50200, Thailand; 5 Center for Neuroscience and Department of Pharmacology, Faculty of Science, Mahidol University, Bangkok, Thailand

**Keywords:** melatonin, aging, hippocampus, sirtuin1, FOXO1, melatonin receptor

## Abstract

Sirtuin1 (SIRT1) and forkhead box transcription factor O subfamily 1 (FOXO1) play vital roles in the maintenance of hippocampal neuronal homeostasis during aging. Our previous study showed that melatonin, a hormone mainly secreted by the pineal gland, restored the impaired memory of aged mice. Age-related neuronal energy deficits contribute to the pathogenesis of several neurodegenerative disorders. An attempt has been made to determine whether the effect of melatonin is mediated through the SIRT1-FOXO1 pathways. The present results showed that aged mice (22 months old) exhibited significantly downregulated SIRT1, FOXO1, and melatonin receptors MT1 and MT2 protein expression but upregulated tumor suppressor protein 53 (p53), acetyl-p53 protein (Ac-p53), mouse double minute 2 homolog (MDM2), Dickkopf-1 (DKK1) protein expression in mouse hippocampus compared with the young group. Melatonin treatment (10 mg/kg, daily in drinking water for 6 months) in aged mice significantly attenuated the age-induced downregulation of SIRT1, FOXO1, MT1 and MT2 protein expression and attenuated the age-induced increase in p53, ac-p53, MDM2, and DKK1 protein and mRNA expression. Melatonin decreased p53 and MDM2 expression, which led to a decrease in FOXO1 degradation. These present results suggest that melatonin may help the hippocampal neuronal homeostasis by increasing SIRT1, FOXO1 and melatonin receptors expression while decreasing DKK1 expression in the aging hippocampus. DKK1 can be induced by the accumulation of amyloid β (Aβ) which is the major hallmark of Alzheimer's disease.

## Introduction

Brain aging is linked to certain types of neurodegenerative diseases and has become critical for the identification of new therapeutic targets. It is currently known that the pathological processes in aging brain are associated with molecules and signaling pathways that sense and influence energy metabolism, e.g., sirtuins (SIRT1), forkhead box transcription factor O subfamily (FOXOs). SIRT1 and the FOXOs are the focus of many brain aging studies because of their interaction with multiple target genes. The protective mechanisms of SIRT1 are closely related to many substrates. FOXOs, one of SIRT1 targets, play a vital role in cellular homeostasis. FOXO1 is highly expressed in the hippocampus and plays an important role in modulating hippocampal neuronal homeostasis (Abbas et al., 2009[1]).

Tumor suppressor protein 53 (p53) is another SIRT1 target that plays a vital role in the apoptosis pathway during aging. SIRT1 suppresses apoptosis through deacetylated p53 and p53-related factors in many senescence models (Arunachalam et al., 2014[2]; Engel and Mahlknecht, 2008[19]; Rahman et al., 2012[47]; Rodella et al., 2013[50]). In addition, the p53 pathway controls FOXOs expression. The mouse double minute 2 homolog (*Mdm2*) and the E3 ubiquitin-protein ligase are important negative autoregulators of p53. MDM2 binds to FOXO1 and FOXO3A and promotes their ubiquitination and subsequent degradation (Chung et al., 2015[13]; Fu et al., 2009[21]; Huang and Tindall, 2011[25]). The Wnt/β-catenin pathway regulates FOXO1 activities. In the nucleus, β-catenin acts as a transcriptional co-regulator with FOXOs, binding to specific DNA-binding transcription factors. Therefore, β-catenin enhances *FOXO1* transcriptional activity (Cadigan, 2008[5]). Cellular oxidative stress simultaneously increases binding between β-catenin and FOXOs, which triggers stress response cascades (Purro et al., 2014[46]).

Melatonin, a hormone mainly secreted by the pineal gland, is another anti-aging agent that could potentially be used to treat neurodegenerative diseases (Hardeland, 2013[24]; Karasek, 2004[28], 2007[27]). Our recent study showed that melatonin restored the impaired memory and attenuated the alteration of the amyloid precursor protein (APP) proteolytic processing enzyme (α- and β-secretases) expression in the hippocampus of aged mice (Mukda et al., 2016[40]). Melatonin preserved the relative protein levels of SIRT1 in the senescence-accelerated mice (SAMP8) (Cristofol et al., 2012[14]; Gutierrez-Cuesta et al., 2008[23]; Tajes Orduna et al., 2009[55]) and in the hippocampus of total sleep-deprived rats (Chang et al., 2009[12]). Age-related neuronal energy deficits contribute to the pathogenesis of several neurodegenerative disorders, such as Alzheimer's disease. Understanding of how melatonin together with molecular, cellular and systemic energy metabolisms may lead to greater understanding of anti-aging mechanisms to increase life span under healthy condition. The present study aimed to answer 3 questions. Firstly, whether melatonin promoted FOXO1 expression and activities via an increase in SIRT1 expression, secondly, whether melatonin promoted FOXO1-β-catenin activities through decreasing DKK1 and thirdly, we also asked whether melatonin increased FOXO1 expression via a p53-MDM2 interaction and decreased the degradation of FOXO1.

## Material and Methods

### Animals

Male mice (Mlac:ICR, outbreed, *Mus musculus*) used in the present experiment were obtained from the National Experimental Animals Center of Mahidol University, Salaya Campus, Thailand. All animal procedures were approved through the Laboratory Animal Care and Use Committee of Mahidol University. We certify that we carried out in accordance with the National Institute of Health Guide for the Care and Use of Laboratory Animals (NIH Publications No. 80-23) revised 1996. All efforts were made to minimize the number of animals used and their suffering. The animals had free access to water and food and were maintained in a 12 h light/dark cycle (light on at 7.00 a.m.). The mice were divided into three treatment groups: (i) control young adult mice (2 months of age, body weight = 30-35 g), (ii) aged group (22 months old mice, body weight = 45-50 g), and (iii) aged mice + melatonin. Melatonin (Sigma Aldrich, St Louis, MO) solution was freshly prepared daily. The long term melatonin-treated mice received melatonin in drinking water from 16 to 22 months of age (for a period of 6 months) according to our previous study protocol (Mukda et al., 2016[40]). The weight of each mouse and the amount of water containing melatonin intake for each mouse were measured. The daily melatonin intake for each mouse was approximately 10 mg/kg (Cavallo and Hassan, 1995[11]; Daya et al., 2001[15]; Moussaoui and Bendriss, 2014[39]; Mukda et al., 2016[40]). Control mice were continued on normal drinking water. At 22 months of age, the mice were sacrificed, the brains were isolated and the hippocampus was dissected and stored at -80 °C until further use.

### Semi-quantitative reverse transcription PCR (RT-PCR)

Total RNA was isolated using the TRIzol^TM^ reagent (Invitrogen, Life Technologies, Carlsbad, CA, USA) according to the manufacturer's protocol. RNA samples with sufficient purity A260/A280 ratios at 1.8-2.0 were used for cDNA synthesis. Two microgram of total RNA was reverse transcribed by using reverse transcription kit (Promega, Madison, WI, USA) with an MJ Mini™ Gradient thermal cycler (Bio-Rad, Hercules, CA, USA). PCR amplification was carried out using primer sets specific for *SIRT1, FOXO1*, *p53*, *Mdm2*, and *Gapdh*. The primer sequences are shown in Table 1(Tab. 1). PCR amplification was carried out at 95 °C for 40 s, 55 or 60 °C for 45 s, and 72 °C for 50 s, which was repeated for 30-35 cycles followed by incubation at 72 °C for 10 min. The amplified products were electrophoresed on 1 % agarose gels in TBE buffer (89 mM Tris-base pH 7.6, 89 mM boric acid, 2 mM EDTA). The gel was stained with RedSafe^TM^ Nucleic Acid Stained (iNtRON, South Korea). The quantity and base pair size of the PCR products were estimated relative to 100 bp DNA ladder marker (Thermo Fisher Scientific Inc., Waltham, Massachusetts, USA). The gel images were photographed with an image analysis system (GelDoc 1000; Bio-Rad, Hercules, CA, USA). Using the Gene Tools analysis software (Syngene, Cambridge, UK), the intensities of specific PCR bands were quantified in relation to the *Gapdh* bands that were amplified from the same cDNA.

### Western blot analysis

Protein extracts were prepared from tissues samples mixed with lysis buffer (150 mM NaCl, 50 mM Tris-base, 1 mM phenyl methanesulfonyl fluoride, 1 mM EDTA, 1 % Triton X-100, 0.5 % sodium deoxycholate, 0.1 % SDS, 1 % protease inhibitor and 1 % phosphatase inhibitor) and sonicated twice for 10 s each. The lysate was then centrifuged at 12,000 x g for 15 min at 4 °C, and the supernatant was collected and used for Western blot analysis. Protein concentration was determined using the Bradford protein assay. Samples were denatured after adding 2X SDS sample buffer. Samples were electrophoresed on 10-12 % SDS-PAGE gels and transferred onto PVDF membranes. The membranes were blocked with blocking buffer for 1 h at room temperature and then incubated overnight at 4 °C with different antibodies as shown in Table 2(Tab. 2); mouse monoclonal antibody against SIRT1 (1:2,000), rabbit polyclonal antibody against FOXO1 (1:2,000), rabbit polyclonal antibody against DKK1 (1:2,000), mouse monoclonal antibody against MDM2 (1:2,000), rabbit polyclonal antibody against p53 (1:2,000), and rabbit polyclonal antibody against acetylated p53 (Lys 379) (1:2,000). All antibodies were diluted using Can Get Signal™. After washing three times with TBST, the membranes were incubated with anti-mouse IgG antibody (1:5,000), or anti-ra:bit IgG antibody (1:5,000). The membranes were washed three times with Tris-Buffered Saline and Tween 20 (TBST) and processed for chemiluminescence detection using ECL Plus™. Western blotting detection reagents were added, and the membrane was exposed using the Azure c300 Chemiluminescent Western Blot Imaging System™ (Azure Biosystems, Inc., Dublin, CA, USA). The immunoblot band densities were quantified using a densitometer with the Scion image program (National Institutes of Health, Bethesda, MD).

### Statistical analysis

The data are expressed as means ± S.E.M. Significance was assessed using one-way analysis of variance (ANOVA), followed by Tukey-Kramer post-hoc tests, using GraphPad Prism version 5. P-values less than 0.05 were considered significant.

## Results

### SIRT1 and FOXO1 protein and mRNA expressions in the aged hippocampus

**Figure 1:**
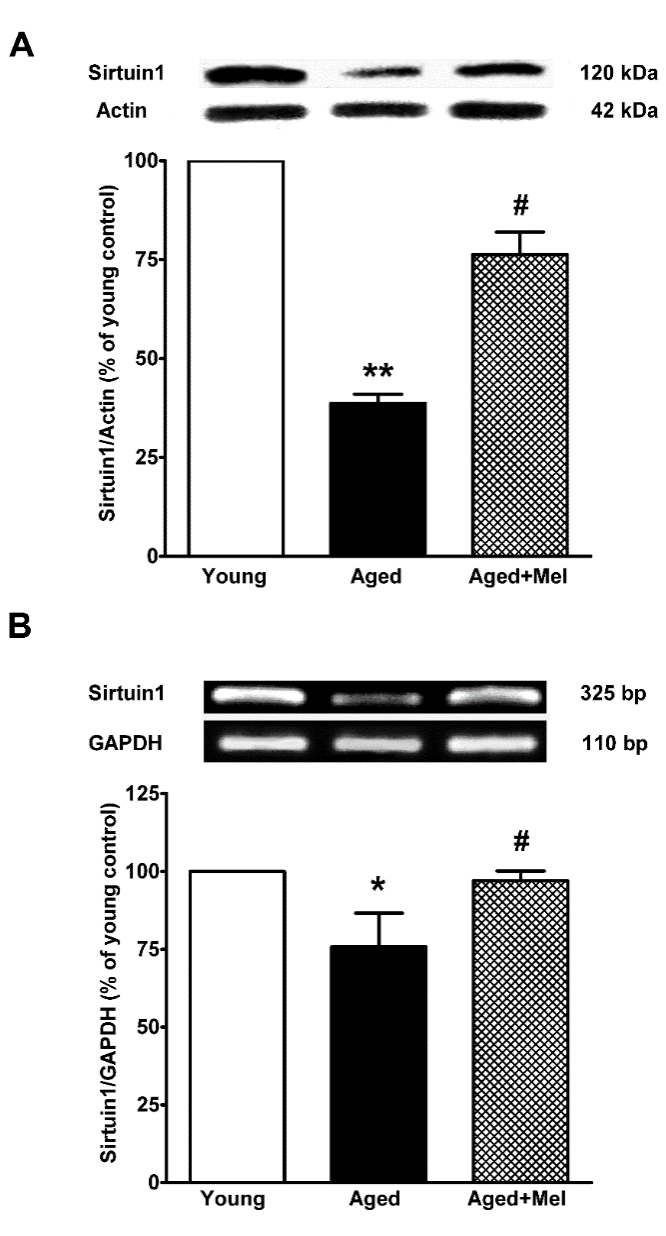
The effect of melatonin on sirtuin1 (SIRT1) protein (A) and mRNA (*B*) expression in the aging mouse hippocampus. SIRT1 protein expression in the hippocampus was compared between young and aged mice. Aged mice were treated with drinking water or with melatonin (Mel) at 10 mg/kg/day for 6 months. Representative bands from the different groups are shown. The bands from the semi-quantitative PCR were normalized to *Gapdh*, whereas the band densities from the Western blots were normalized to actin. The ratios were calculated as a percentage of the respective value of the control group. The values represent means ± SEM (n=4 for each group). (* and ** denote significant differences with p < 0.05 and p < 0.01, respectively, compared with young control group, and # denotes a significant difference with p < 0.05 compared with the aged control group).

**Figure 2:**
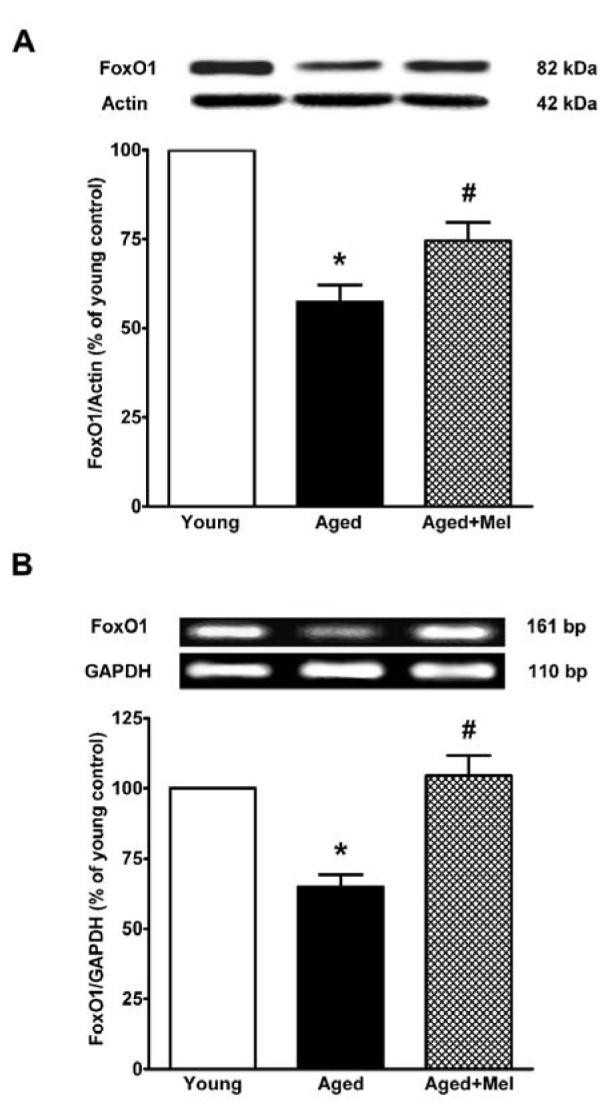
The effect of melatonin on FOXO1 protein (A) and mRNA (B) expression in the aging mouse hippocampus. The protein expression of FOXO1 was compared between young and aged mice. Aged mice were treated with drinking water or with melatonin (Mel) at 10 mg/kg/day for 6 months. Representative bands from the different groups are shown. The bands from the semi-quantitative PCR were normalized to *Gapdh*, whereas the band densities from the Western blots were normalized to actin. The ratios were calculated as a percentage of the respective value of the control group. The values represent means ± SEM (n=4 for each group). (* denotes a significant difference with p < 0.05 compared with the young control group, and # denotes a significant difference with p < 0.05 compared with the aged control group).

SIRT1 protein and mRNA expression were significantly decreased in the aged hippocampus when compared to the expression in young mice. The melatonin-treated group significantly increased SIRT1 protein and mRNA expression in aged mice compared with the aged untreated group (Figures 1A, B(Fig. 1)). FOXO1 protein and mRNA expression were significantly decreased in aged mice. The melatonin-treated group significantly upregulated FOXO1 protein and mRNA expression in the hippocampus of aged mice when compared with the aged control group (Figures 2A, B(Fig. 2)). 

### p53 protein and mRNA expressions in the aged hippocampus

**Figure 3:**
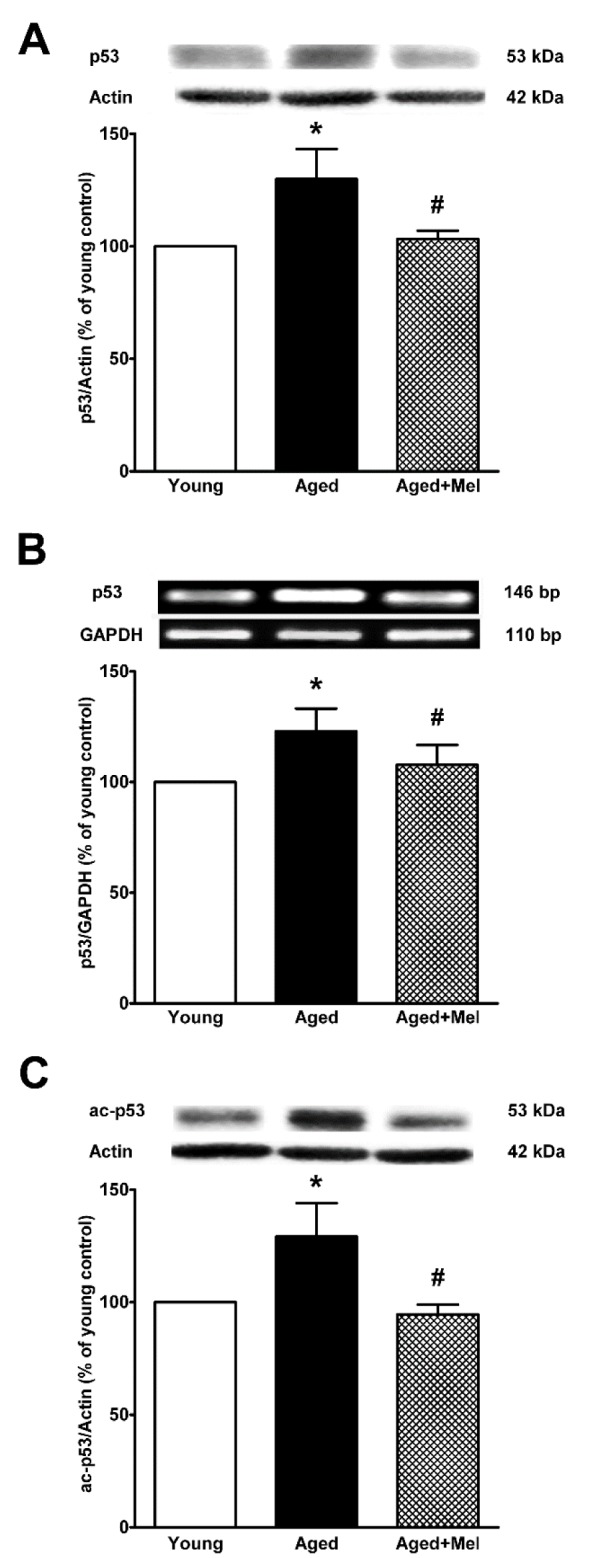
The effect of melatonin on p53 protein expression (A), p53 mRNA expression (B) and the acetylated form of p53 protein expression (C) in the aging mouse hippocampus. Aged mice were treated with drinking water or melatonin (Mel) at 10 mg/kg/day for 6 months. Representative bands from the different groups are shown. The bands from the semi-quantitative PCR were normalized to *Gapdh*, whereas the band densities from the Western blots were normalized to actin. The ratios were calculated as a percentage of the respective value of the control group. The values represent means ± SEM (n=4 for each group (* denotes a significant difference with p < 0.05 compared with the young control group, and # denotes a significant difference with p < 0.05 compared with the aged control group).

Because the p53-MDM2 regulatory pathway can modulate FOXO1 expression, we determined the effect of melatonin on p53 expression in the hippocampus of aged mice. Protein and mRNA expression of p53 in aged hippocampus significantly increased when compared to the young control group. Melatonin significantly decreased p53 protein and mRNA expression levels compared with those seen in the aged control group (Figures 3 A, B(Fig. 3)). Because p53 is a SIRT1 target, we explored SIRT1 activity by detecting the acetylated form of p53. Ac-p53 protein expression in aged mice was higher than that seen in the young control group. Melatonin significantly attenuated the increase in Ac-p53 expression in aged mice compared to the aged control group (Figure 3 C(Fig. 3)).

### MDM2 protein and mRNA expressions in the aged hippocampus

**Figure 4:**
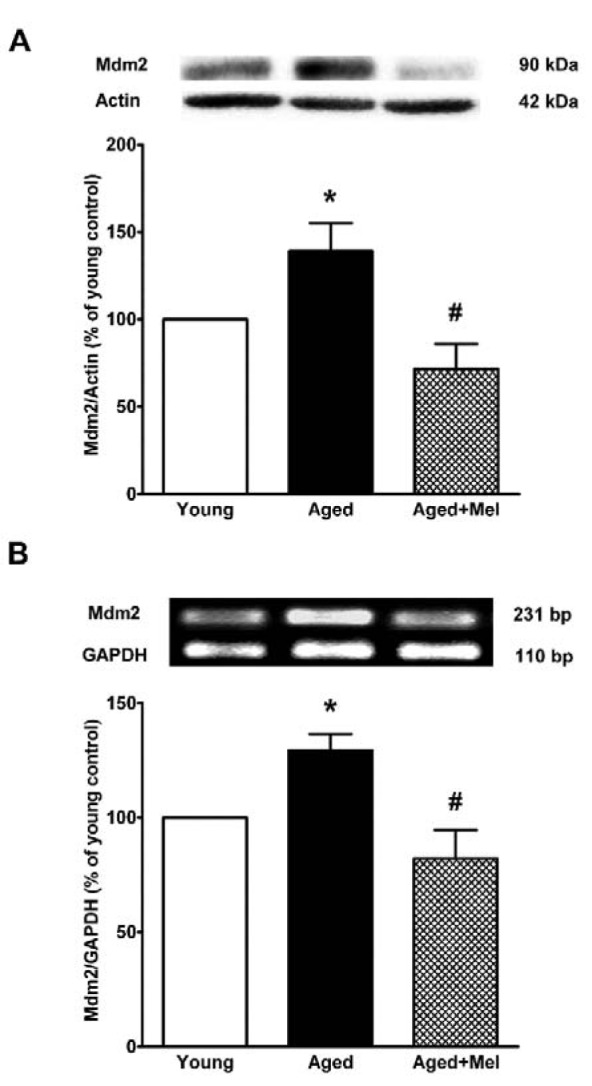
The effect of melatonin on MDM2 protein (A) and mRNA (B) expression in the aging mouse hippocampus. Aged mice were treated with drinking water or melatonin (Mel) at 10 mg/kg/day for 6 months. Representative bands from the different groups are shown. The bands from the semi-quantitative PCR were normalized to *Gapdh*, whereas the band densities from the Western blots were normalized to actin. The ratios were calculated as a percentage of the respective value of the control group. The values represent means ± SEM (n=4 for each group). (* denotes a significant difference with p < 0.05 compared with the young control group, and # denotes a significant difference with p < 0.05 compared with the aged control group).

We determined whether MDM2, an ubiquitin E3 ligase for p53, can degrade FOXO1. Protein and mRNA expression levels of *Mdm2* in the aged hippocampus were significantly increased when compared to the expression seen in the hippocampus of young mice. Melatonin treatment of the aged group significantly decreased MDM2 protein and mRNA expression compared with the expression levels in the aged control group (Figures 4A, B(Fig. 4)). The result suggested that the ubiquitination of FOXO1 in the aged hippocampus might be due to the activity of MDM2. 

### DKK1 protein and mRNA expressions in the aged hippocampus

**Figure 5:**
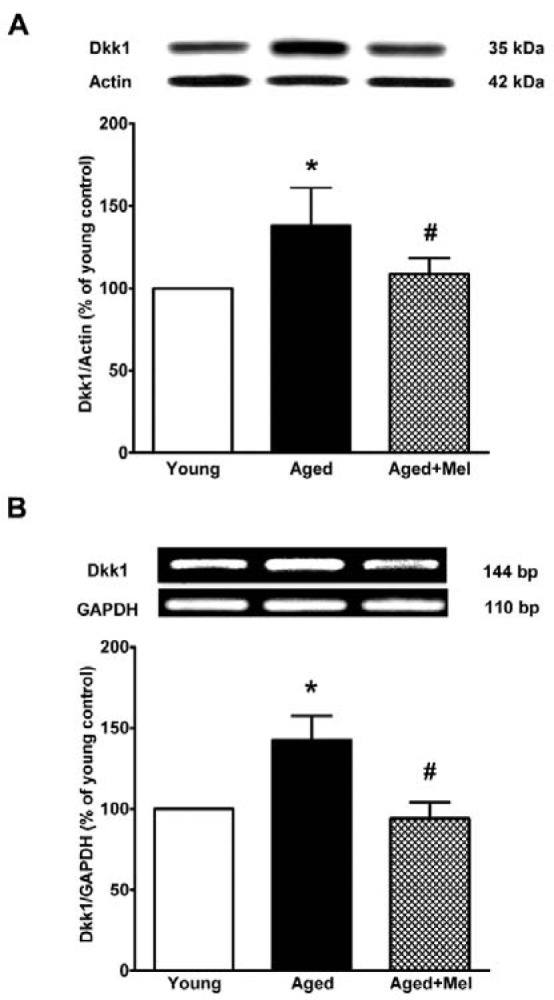
The effect of melatonin on DKK1 protein (A) and mRNA (B) expression in the aging mouse hippocampus. Aged mice were treated with drinking water or melatonin (Mel) at 10 mg/kg/day for 6 months. The representative bands from the different groups are shown. The bands from the semi-quantitative PCR were normalized to *Gapdh*, whereas the band densities from the Western blots were normalized to actin. The ratios were calculated as a percentage of the respective value of the control group. The values represent means ± SEM (n=4 for each group). (* denotes a significant difference with p < 0.05 compared with the young control group, and # denotes a significant difference with p < 0.05 compared with the aged control group).

DKK1, a specific negative modulator of the Wnt pathway, was investigated in this study to determine whether the melatonin-induced increase in FOXO1 activity occurs via the Wnt/β-catenin pathway. The results showed that DKK1 protein and mRNA levels in the aged hippocampus were significantly increased when compared to those seen in the young group. The melatonin-treated group significantly decreased DKK1 protein and mRNA expression when compared to the levels seen in the aged control group (Figures 5A, B(Fig. 5)). 

### MT1 and MT2 melatonin receptors proteins in the aged hippocampus

**Figure 6:**
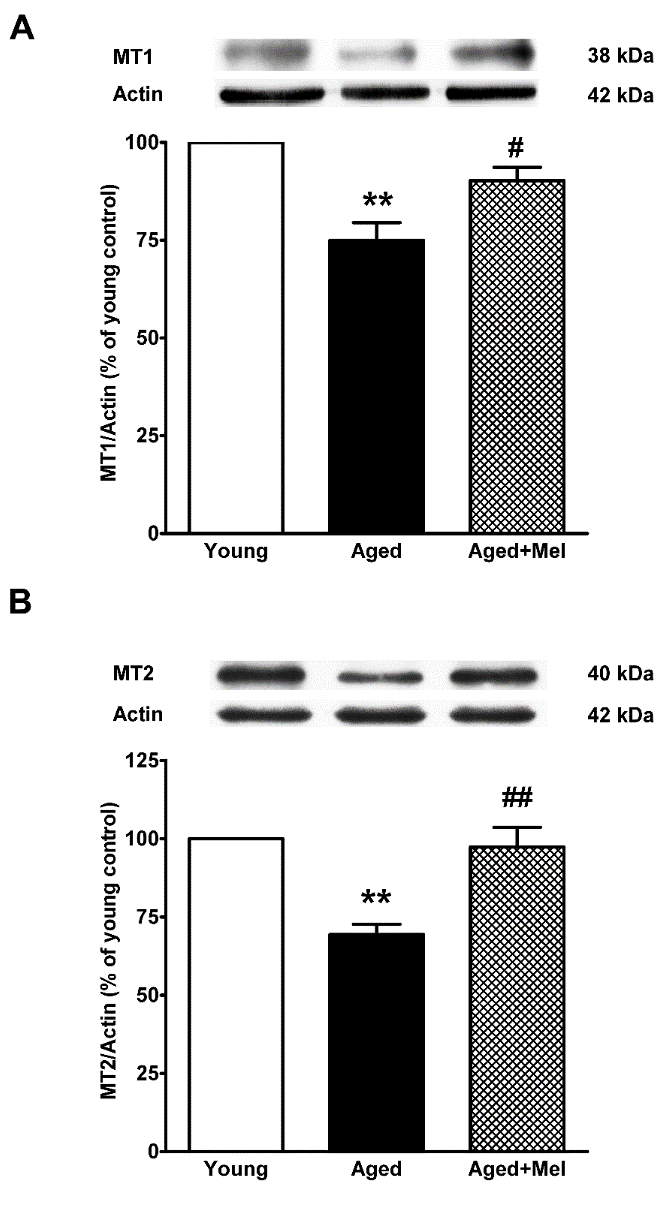
The effect of melatonin on MT1 (A) and MT2 (B) expression in the aging mouse hippocampus. Aged mice were treated with drinking water or melatonin (Mel) at 10 mg/kg/day for 6 months. The representative bands from the different groups are shown. The band densities from the Western blots were normalized to actin. The ratios were calculated as a percentage of the respective value of the control group. The values represent means ± SEM (n=4 for each group). (** denotes significant difference with p < 0.01, compared with the young control group, # and ## denote significant difference with p < 0.05 and p < 0.01 compared with the aged control group).

The levels of melatonin receptors MT1 and MT2 were examined (Figures 6A, B(Fig. 6)). In aging hippocampus, MT1 and MT2 levels were significantly downregulated when compared to those seen in the young group. Melatonin was able to significantly restore the reduction of melatonin receptors in the aged group. 

## Discussion

In the present study it has been shown that SIRT1 was downregulated in the aged hippocampus, while melatonin treatment for 6 months restored SIRT1 expression. Previous studies demonstrated that melatonin increased SIRT1 expression in a variety of models (Chang et al., 2009[12]; Tajes et al., 2009[54]). Melatonin protected neurons by preserving protein levels of SIRT1 in the dentate gyrus of aged rats (Kireev et al., 2013[29]), a total sleep-deprived model (Chang et al., 2009[12]), SAMP8 mice (Cristofol et al., 2012[14]; Gutierrez-Cuesta et al., 2008[23]; Tajes et al., 2009[54]) and neonatal rats subjected to hypoxia-ischemia (Carloni et al., 2014[10]). SIRT1 is NAD^+^-dependent deacetylase that senses changes in intracellular NAD^+ ^levels (Canto and Auwerx, 2012[6]), the supportive effect of melatonin on SIRT1 may act via the nicotinamide adenine dinucleotide (NAD) system. Melatonin, an effective antioxidant, preserves NAD levels under oxidative stress. When melatonin was incubated with the PC12 cell line in an oxidative environment, the oxidation was reduced and melatonin donated an electron to the NAD radical, resulting in the generation of NAD (Tan et al., 2005[56]).

We found that FOXO1 was downregulated by aging, and the reduction could be prevented by melatonin administration. FOXO1 was predominantly expressed in the hippocampus and declined during aging (Zemva et al., 2012[61]). Melatonin may increase FOXO1 expression through the PI3K/Akt pathway. In an aged neuronal cell culture, melatonin increased Akt activation, leading to GSK3β inhibition and an increase in FOXO1 phosphorylation (Tajes et al., 2009[54]). Melatonin had protective effects against brain injury through activating Akt and its downstream targets in a middle cerebral artery occlusion model (Koh, 2008[31]). Melatonin increased the interaction of phosphorylated-FOXO1 (p-FOXO1) and 14-3-3 in a kainic acid-induced hippocampal excitotoxicity model (Lee et al., 2006[34]) and in focal cerebral ischemia in rats (Zheng et al., 2014[63]). The acetylation of FOXO1 decreased DNA binding and FOXOs activity (Bousiges et al., 2010[3]; Wang et al., 2012[59]). Melatonin upregulated the expression of SIRT1 and downregulated the expression of ac-FOXO1 in a cecal ligation brain injury model (Zhao et al., 2015[62]). Melatonin not only promotes FOXOs activities via SIRT1 deacetylation but also acts through CREB-binding protein (CBP) and p300. Melatonin suppressed p300 histone acetyltransferase (HAT) activity and p300 HAT-mediated NF-κB acetylation in the human vascular smooth muscle cell line CRL1999 (Shi et al., 2012[52]).

In the present study, melatonin treatment in aged animal decreased p53, acetyl-p53 compared with aged mice. This ameliorated the FOXO1 degradation process. Melatonin decreased p53 both in *in vitro* and *in vivo *studies (Gutierrez-Cuesta et al., 2008[23]). SIRT1 expression was lower in SAMP8 mice compared with SAMR1 mice, and these changes were prevented by melatonin (Gutierrez-Cuesta et al., 2008[23]). In a neuronal cell culture, melatonin treatment inhibited the Ataxia telangiectasia mutated (ATM) protein. ATM is a serine/threonine protein kinase that phosphorylates several key proteins that initiate the activation of the DNA damage checkpoint, including p53 and MDM2. Moreover, melatonin administration activated the prosurvival protein Akt, which resulted in GSK-3β inhibition and an increase in p-FOXO1 (Tajes et al., 2009[54]). 

p53 activation is mainly regulated by MDM2 in the nucleus. MDM2 also acts as E3 ubiquitin ligase, promoting proteasome-dependent p53 degradation; therefore, p53 and MDM2 regulate each other in an autoregulatory loop. Melatonin drastically downregulated *Mdm2* gene expression and inhibited MDM2 shuttling into the nucleus of MCF7 breast cancer cells. Because PI3K/Akt-mediated MDM2 phosphorylation on serine 166 is essential to enable MDM2 entry in the nucleus, melatonin slowed down the activity of the PI3K/AkT pathway, leading to a reduction in MDM2 levels. In addition, the inhibition of Akt-mediated MDM2 phosphorylation prevents the nuclear translocation of MDM2 and consequently impairs its ability to target and degrade p53 (Proietti et al., 2011[44], 2014[45]). Both MDM2 and p53 graphs showed the same trend of expression in the present results, since P53 and MDM2 regulate each other in an autoregulatory feedback loop. If the expression of MDM2 or p53 is out of the autoregulatory manner, this will trigger the DNA damage (Pant et al., 2013[41]).

The neuroprotective effects of melatonin may be due to a decrease in the acetylated form of p53. The acetylation of p53 on some residues such as lysine373, enhanced the stabilization/activity of p53 and increased the susceptibility of cells to stress. Acetylation of p53 was altered in the hippocampus following global cerebral ischemia (Raz et al., 2011[48]). In addition, the acetylated form of p53 attenuated mdm2-mediated ubiquitination, possibly through inducing a protein conformational change (Ito et al., 2002[26]; Li et al., 2002[36]).

To investigate whether melatonin affects Wnt/β-catenin signaling, we determined the expression of the specific inhibitor Dickkopf-1 (DKK1). We found that DKK1 protein and mRNA levels in the aged hippocampus were higher than those in the young group, and melatonin treatment lowered the level of DKK1 protein and mRNA. DKK1 is a specific inhibitor of the Wnt pathway, and antagonizes signaling by binding to low-density lipoprotein receptor-related protein 5/6 (LRP5/6). DKK1 is scarcely found in normal brain, but upregulation of DKK1 precedes neuronal death in models of neurodegenerative diseases (Caricasole et al., 2004[9]; Rosi et al., 2010[51]) and ischemic neuronal death (Cappuccio et al., 2005[7]). Melatonin may decrease DKK1 via downregulation of p53. DKK1 is induced by p53 because the *Dkk1* transcription start site has a p53 responsive element (Caricasole et al., 2004[9]; Wang et al., 2000[58]), whereas DKK1 and Wnt inhibitory factor-1 (WIF1) significantly activated the transcription of p53 in ovarian carcinoma cells (Ko et al., 2014[30]). In addition, mature neurons exposed to DKK1 showed a decrease in the number of functional synaptic vesicle recycling sites, and DKK1 promoted the dispersion of pre- and postsynaptic proteins, indicating a loss of synapses (Purro et al., 2014[46]). The inhibition of the Wnt pathway in neurospheres by retroviral overexpression of *Dkk1* robustly increased gliogenesis at the expense of neurogenesis (Kunke et al., 2009[32]). Aging rat hippocampus exhibited an upregulation of DKK1, while exercise could reverse this change in expression.

Several lines of evidence have suggested a pathophysiological relationship between reduction in melatonin and melatonin receptors with aging and neurodegenerative diseases. Our result here showed that MT1 and MT2 declined during aging while melatonin supplement was able to reverse this change. Melatonin transmits its action via the specific high affinity G-protein couple receptors (Reppert et al., 1994[49]) which are expressed in several areas of the brain, including hippocampus (Mazzucchelli et al., 1996[38]). Melatonin exerts its action on neurogenesis via the membrane-bound receptor both in the subventricular zone (Sotthibundhu et al., 2010[53]) and rat hippocampus (Tocharus et al., 2014[57]). In this study, we found an increase in MT1 and MT2 protein levels after melatonin treatment, which is consistent with our previous studies in cultured adult subventricular zone neurospheres (Sotthibundhu et al., 2010[53]) and in cultured adult hippocampal precursor cells pretreated with dexamethasone (Ekthuwapranee et al., 2015[18]). Activation of MT1 via Gαi-coupled melatonin receptors has been shown to produce the inhibitory responses in the cAMP signal transduction cascade, resulting in decreases in PKA activity and in CREB phosphorylation (Brydon et al., 1999[4]; Dubocovich et al., 2003[17]; Li et al., 1998[35]). Moreover, through the dissociation of βγ subunits of Gi, melatonin activates MAP kinases as well as the Ras-MEK pathway to stimulate ERK phosphorylation (Carbajo-Pescador et al., 2011[8]; Pearson et al., 2001[42]; Witt-Enderby et al., 2003[60]). Activation of the MT2 can also result in decreases in cGMP, decreases cAMP formation and PI hydrolysis via Gαi subunit activation (Dubocovich et al., 2010[16]; Markowska et al., 2004[37]). Melatonin acted through MT1 and MT2 receptors to activate hypothalamic Akt/PKB and protein kinase B to suppress hepatic gluconeogenesis in rats (Faria et al., 2013[20]).

Aging decreases the Wnt pathway activities. Melatonin may help to enhance the Wnt pathway through different pathways. For example; melatonin decreased reactive oxygen species (ROS) and decreased GSK-3β. Increases in GSK-3β activity during aging inactivate β-catenin through phosphorylation. Melatonin increased phosphorylated GSK-3β in primary cultures of cerebellar granule neurons (Tajes et al., 2009[54]), in a SAMP8 mice aging model (Gutierrez-Cuesta et al., 2007[22]) and in an Alzheimer's animal model (Kwon et al., 2010[33]; Peng et al., 2013[43]). Melatonin may act through PI3K-Akt-GSK3β pathway via MT1 and MT2 receptors. In addition, melatonin decreases some factors that are related to neurodegenerative diseases, such as amyloid β (Aβ) since Aβ accumulation induces Dkk1 production.

## Conclusion

In summary, the current study suggested possible mechanisms of the neuroprotective effects of melatonin on the FOXO1 pathway during aging. Melatonin significantly upregulated SIRT1, FOXO1, MT1 and MT2 expression but downregulated p53, ac-p53 and MDM2, the ligase enzyme that ubiquitinates FOXO1. Melatonin may increase FOXO1 expression via downregulation of the p53-MDM2 pathway. The expression of DKK1, a specific inhibitor of the canonical Wnt pathway, was downregulated by melatonin. Melatonin may promote FOXO1 activities by inducing β-catenin, the co-transcription factor of FOXO1. 

## Acknowledgements

This study was supported by the Thailand Research Fund (TRF) (DPG5780001 and IRG5780009) and a Mahidol University Research Grant to PG.

## Figures and Tables

**Table 1:**
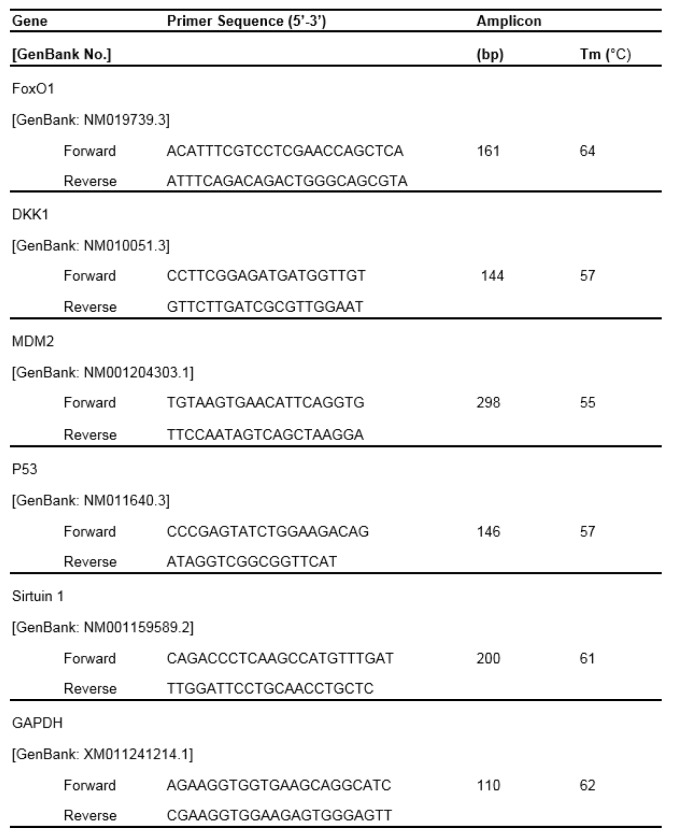
Primer sequences for semi-quantitative RT-PCR

**Table 2:**
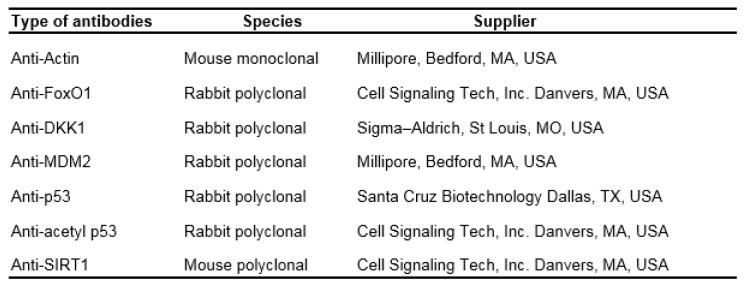
The antibodies used for Western blotting
